# *Zangfu zheng *(patterns) are associated with clinical manifestations of *zang shang* (target-organ damage) in arterial hypertension

**DOI:** 10.1186/1749-8546-6-23

**Published:** 2011-06-17

**Authors:** Alexandre Bastos Luiz, Ivan Cordovil, José Barbosa Filho, Arthur Sá Ferreira

**Affiliations:** 1 Amarina Motta School Clinic, Augusto Motta University Center, Av. Paris 72, Bonsucesso, Rio de Janeiro, BR CEP 21041-020, Brazil; 2 Division of Arterial Hypertension, National Institute of Cardiology, Rua das Laranjeiras 374, Laranjeiras, Rio de Janeiro, BR CEP 22240-006, Brazil; 3 Department of Cardiology, Medical School, Gama Filho University, Rua das Laranjeiras 374, Laranjeiras, Rio de Janeiro, BR CEP 22240-006, Brazil; 4 Program of Rehabilitation Science, Centro Universitário Augusto Motta, Praça das Nações 34, Bonsucesso, Rio de Janeiro, BR CEP 21041-010, Brazil

## Abstract

### Background

Hypertension is a clinical condition that manifests target-organ damage (TOD) with symptoms. This study investigates the association between *Zangfu *patterns and symptomatic manifestations of TOD.

### Methods

Datasets with manifestations of *Zangfu *patterns (Liver-fire blazing upwards; Kidney-yin deficiency and Liver-yang rising; obstruction of phlegm and dampness of Heart/Liver/Gallbladder; *qi *and blood deficiency leading to Liver-yang rising; Kidney-yin/yang deficiency) and TODs (cerebrovascular, heart and kidney) were compiled from literature. The Pattern Differentiation Algorithm was used to test and to determine diagnostic accuracy with these datasets. A questionnaire was developed from datasets and applied to 43 subjects newly diagnosed with hypertension. Pattern differentiation was performed and the results were statistically analyzed for association between descriptions of patterns and TOD.

### Results

The observed diagnostic accuracy, sensitivity and specificity were 98.0%, 96.2% and 99.8% respectively. Similarity between patterns and TOD datasets was mostly negligible. Twelve manifestations demonstrated high prevalence, namely red tongue (81.4%), headache (72.1%), irritability (67.4%), palpitation (60.5%), blurred vision, insomnia and mental fatigue (58.1%), frequent nocturnal urination, numbness in feet and hands, shortness of breath (55.8%), and heavy limbs sensation, wiry pulse (51.2%). No significant association was found between blood pressure variables (systolic, diastolic, mean, pulse pressure) and manifestations.

### Conclusion

*Zangfu *patterns are associated with clinical manifestations of TOD. Manifestations associated patterns indicate morbid conditions to be secondary to hypertension rather than simple blood pressure.

## Background

### Morbidity research on diseases and patterns

Ancient Chinese medicine literature [1-4] is rich in records of patterns, the Chinese medicine nosological counterpart of disease. Morbidity studies based on Chinese medicine clinical records enhanced practitioner development and training that lead to improved patient care, research programs, public policy and evidence-based commissioning [5,6].

In contemporary Chinese medicine literature [7-12], diseases were assigned to patterns based on matched 'signs and symptoms' (*ie *manifestations) to integrate both medical practices. For instance, studies were conducted in the last two decades for cervical spine cancer (254 cases) [13], frequently recurring cystitis (61 women) [14], hepatocyrrhosis (223 cases [15] and 147 cases [16]), and gastric cancer (767 cases) [17]. Morbidity research of disease-related patterns was advised to focus on public health disorders such as cardiovascular diseases, the principal cause of death in modern society [18].

### Chinese medicine patterns in cardiovascular diseases

Morbidity studies were conducted for variant *angina pectoris *(175 cases) [19], stable *angina pectoris *(251 cases) [20] and acute ischemic stroke (1246 cases) [21]. Despite the worldwide high prevalence of hypertension as the major risk factor for cardiovascular diseases [18], only five Chinese medicine morbidity studies on it were found in literature. As Chinese medicine diagnosis could improve efficacy and/or diminish adverse effects of antihypertensive agents [22], the morbidity of patterns in hypertension must be studied.

Kalish *et al*. [23] reported the Stop Hypertension with the Acupuncture Research Program trial (a pilot randomized clinical trial on the efficacy of acupuncture in treating essential hypertension), which was expected to find *Zangfu *patterns in hypertension. A randomized controlled trial [24] on acupuncture treatment for hypertension enrolled 192 patients and the frequency of *Zangfu *patterns was recorded. However, no data related to observed manifestations were given and no association was investigated between clinical findings (*eg *blood pressure) and patterns. Flachskampf *et al*. [25] randomized the allocation of 160 outpatients with uncomplicated hypertension in a single-blind fashion to a 6-week course of acupuncture intervention; however, they did not report descriptive statistics on patterns or manifestations or association analysis. Chu *et al*. [26] reported 59 cases of hypertension classified according to whether or not abundant phlegm-dampness was presented for analysis of proteome. Again, no analysis was conducted to explore the frequency distribution of patterns or its manifestations. Gu *et al*. [27] investigated the frequency distributions of patterns in 477 untreated subjects with hypertension and did not find statistical significance in the frequency distributions of patterns within blood pressure levels, age or body mass index (BMI). This heterogeneity of analysis regarding patterns in subjects with hypertension led to the reports of opposite results of acupuncture treatment for lowering mean 24-hour ambulatory blood pressures.

### Diagnosis and prognosis of hypertension in Chinese medicine and conventional medicine

As hypertension may be symptomless until late in its course, previous guidelines for management of hypertension advised that its diagnosis should be based on multiple systolic (SBP) and diastolic blood pressure (DBP) measurements (≥ 140 and 90 mmHg respectively) taken on separate occasions over time [28]. A recent study indicated that both family and clinical histories would be required for prognosis in patients with high blood pressure [29]. Current knowledge of hypertension emphasizes the role of structural changes in microcirculation (such as arteriolar rarefaction [30,31]) in hypertension pathogenesis and hypertension-related organ damage [32]. Concomitantly, hypertrophied or remodeled medium-sized vessels [33] and stiffened large arteries [34] are the basis of hypertension-induced organ damage in the brain (and eyes), heart or kidneys [32]. In general, changes in blood flow and pressure are not significant until approximately 50% of the vessel diameter is obstructed [35]. Thus, hypertensive patients' manifestations may indicate the progression or worsening of those target-organ damages (TOD).

On the other hand, Chinese medicine practitioners rely on information collected from the Four Methods (FM, *sizhen*) of examination, namely inspection (*wang*), auscultation and olfaction (*wen*), inquiry (*wen*) and palpation (*qie*), and do not use blood pressure measures for pattern differentiation on patients with hypertension. Contemporary literature on Chinese medicine diagnosis [7-12] and clinical research [23,24,27] assign up to five *Zangfu *patterns to hypertension based only on manifestations. This discrepancy between Chinese medicine and conventional medicine raises the question whether patterns are indeed related to high blood pressure levels or to TOD caused by chronic hypertension. However, no previous study on morbidity of hypertension-related patterns [23-27] has explored the relations between patterns and TOD.

This study investigates the association between *Zangfu *patterns and clinical manifestations of TOD and tests a hypothesis that patterns are associated with TOD as manifestations associated patterns indicate morbid conditions to be secondary to hypertension rather than simple blood pressure.

## Methods

### Study design

**Figure 1:**
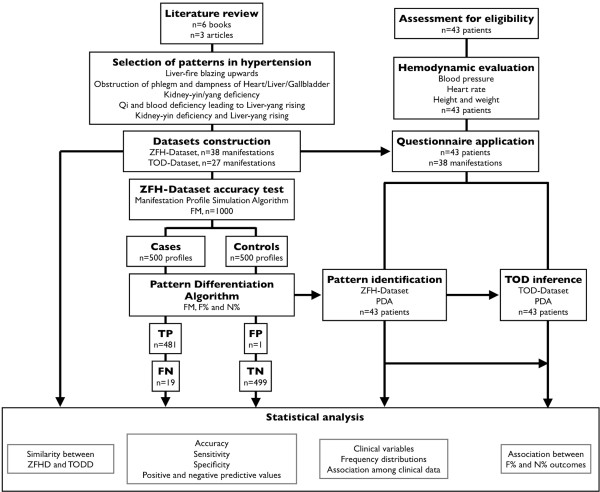
**Study design. **Literature review of *Zangfu *patterns and TOD was performed to generate both datasets and questionnaire. A computer-based method for CM diagnosis was validated for the constructed hypertension dataset. The questionnaire was applied to patients and the results were analyzed test the association between *Zangfu *patterns and TOD.

The design of the present study is described in Figure 1. Literature review of patterns and TOD was performed to generate datasets for Chinese medicine and conventional medicine. The diagnostic accuracy of Pattern Differentiation Algorithm (PDA) for Chinese medicine diagnosis was tested for the constructed hypertension dataset. A questionnaire for clinical assessment of patients was generated from hypertension dataset and applied to subjects with hypertension (according to electronic health records). Statistical analysis was conducted to test the association between descriptions of patterns and TOD. The present study followed the guidelines for the Strengthening the Reporting of Observational Studies in Epidemiology (STROBE) [36].

### Development of pattern dataset and questionnaire

Patterns were collected from contemporary literature [7-12,23,24,27], according to which, five *Zangfu *patterns describing subjects with hypertension are as follows. Liver-fire blazing upwards (*gan huo shang yan*); Kidney-yin deficiency and Liver-yang rising (*shen yin xu gan yang shang yan*); Obstruction of phlegm and dampness of Heart/Liver/Gallbladder (*xin gan dan shi tan bi*); *Qi *and blood deficiency leading to Liver-yang rising (*qi xue xu gan yang shang yan*); and Kidney-yin/yang deficiency (*shen yin yang xu*). These patterns had their respective manifestations annotated according to the FM to compose the widest description of each pattern, namely *Zangfu *hypertension dataset (ZFHD, Table 1). Possible descriptions to distinguish manifestations included its onset, duration, location, progression and severity (Additional file 1).

**Table 1:** Chinese medicine patterns of systemic arterial hypertension

Examination method	Manifestations	Patterns	Target-organs
**Inspection**	Flushed face	A[8,24]	*
	Grease and thick coating	C[8,24]	CVD[51], HD[51]
	Pale tongue	E[8,24]	KD[43,53]
	Peeled tongue	B[24]	*
	Red eyes	A[8]	CVD[40], KD[41]
	Red tongue	A[10,24], B[8,24]	*
	Yellow coating	A[8,24]	CVD[51], HD[51]
**Auscultation-Olfaction**	Aphasia	D[8]	CVD[47,43]
	Shortness of breath	E[8,24]	HD[47,43]
**Inquiry**	Blurred vision	B[8,24]	CVD[47], KD[44]
	Congested feeling in the chest	C[8,24]	HD[47]
	Constipation	A[8,24]	KD[45]
	Convulsions	D[8]	CVD[44,43]
	Dizziness	B[8,24], C[8], E[24]	CVD[47]
	Excessive dreaming	B[8]	*
	Fainting	D[8]	CVD[44]
	Frequent nocturnal urination	E[8,24]	KD[44]
	Headache	A[8,10,24], B[24], D[8]	CVD[47,44], KD[44]
	Heavy limbs sensation	C[8]	CVD[52]
	Impotence	E[8,24]	*
	Insomnia	B[8,24]	CVD[48], HD[48], KD[48]
	Irritability	A[8,10,24]	*
	Mental fatigue	E[8,24]	KD[45]
	Nausea	C[8,24]	CVD[44,43], KD[46]
	Numbness in feet and hands	E[8]	CVD[47,43]
	Numbness in the limbs	B[8,24], C[8]	CVD[47,43]
	Palpitation	C[8]	HD[47,43]
	Severe dizziness	D[8,24]	CVD[47]
	Stroke	D[8]	CVD[47,43]
	Tinnitus	A[10], B[8,24], E[8,24]	CVD[42]
	Vomiting	C[8]	CVD[52], KD[46]
	Weak legs	E[8]	CVD[47,43], KD[44]
**Palpation**	Deep pulse	E[8,24]	*
	Fast pulse	A[8,24], B[8,24]	HD[47]
	Slippery pulse	C[8,24]	*
	Strong pulse	A[8]	*
	Thin pulse	B[8],24, E[8,24]	*
	Wiry pulse	A[8,10,24], B[8,24], C[8], D[24]	*

Chinese medicine classification and description of patterns and respective target-organs related to hypertension.

Legends: A: Liver-fire blazing upwards; B: Kidney-yin deficiency and Liver-yang rising; C: Obstruction of phlegm and dampness of Heart/Liver/Gallbladder; D: Qi and blood deficiency leading to Liver-yang rising; E: Kidney-yin/yang deficiency; CVD: cerebrovascular and eye disease; HD: heart disease; KD: kidney disease. Manifestations not considered as related to specific target-organs were marked as * and were not used for inference about target-organ damage.

The questionnaire was automatically generated from ZFHD by an algorithm as follows. Since patterns may share manifestations (co-occurrence of terms), all patterns in ZFHD were merged and their respective manifestations cited only once. Then, the dataset was submitted to a two-stage processing scheme for intra-pattern and inter-pattern quality control [37,38]. The resulting *Zangfu *hypertension questionnaire (ZFHQ) was composed of 38 manifestations distributed among inspection (*n *= 7; 18.4%), auscultation-olfaction (*n *= 2; 5.3%), inquiry (*n *= 23; 60.5%) and palpation (*n *= 6; 15.8%).

### Development of TOD dataset: Correspondence between patterns and TOD

To analyze the association between descriptions of patterns and TOD, we redistributed the manifestations in ZFHD per target-organ within each examination method to compose the TOD dataset (TODD, Table 1). Most of the signs and symptoms describing patterns were easily recognized as corresponding to cerebrovascular, heart or kidney lesions [29,39-47]; however, some manifestations particularly related to tongue inspection and pulse palpation due to predominant qualitative descriptions were not explored [48]. In this study, the following manifestations were assigned based on the argument that a strong correlation (*r *= 0.74; *P *= 0.001) was found between a thick yellow or gray tongue coating and halitosis resulting from infection with specific bacterial species such as *Solobacterium moorei *which produces high levels of volatile sulfur compounds. Evidence suggested that even low concentrations of those compounds might be toxic and played a role in the link between oral infection and either heart [49] or cerebrovascular disease [50]. Conversely, the peeled (without coat) tongue was found to appear in subjects with healthy periodontal tissues [51]. The pallor observed in anemia, a common feature of chronic kidney failure [42], was better identified at the tongue compared to conjunctivae, palms or nail beds and tongue pallor might rule out and modestly rule in severe anemia [52]. All remaining manifestations were not assigned to TOD and consequently not used in the current analysis. The resulting TODD was composed by 27 manifestations distributed among inspection (*n *= 4; 14.8%), auscultation-olfaction (*n *= 2; 7.4%), inquiry (*n *= 20; 74.1%) and palpation (*n *= 1; 3.7%).

### Subject recruitment

The study was conducted with a sample of patients from National Institute of Cardiology (Rio de Janeiro, Brazil) after the Medical Ethics Committee had approved the protocol (trial register number 0239/02.06.09). Written informed consent was obtained from all subjects in the study. Subjects newly diagnosed with hypertension were prospectively recruited from January 2009 to July 2009 (once a week) and admitted in this study after physical examination. The diagnostic criteria of hypertension were systolic and diastolic arterial blood pressure ≥140 or 90 mmHg respectively, measured in two or more consecutive visits at the outpatient clinic over a period of at least seven days [29]. Patients did not report a prior use of any antihypertensive. Metabolic diseases (such as diabetes mellitus) and secondary hypertension were clinically investigated and ruled out in all subjects. Sample sizes were estimated from formulae [53] designed for studies with correlation coefficient as the outcome. A minimum sample size of 36 cases is required to test for the alternative hypothesis that the correlation coefficient is higher than 0.41 (at least weak association) with α = 5% (significance level) and β = 80% (power of test).

### Clinical variables and data measurement

Forty-three subjects (among which 26 were female) with primary hypertension were enrolled in this study. All procedures were performed between 08:00 and 12:00 in a quiet room with controlled temperature (19-21°C) immediately before the questionnaire interview. Ambulatory blood pressures were measured at the brachial artery (right arm) with a mercury column sphygmomanometer by the same examiner. The first and the fifth Korotkoff's phases were used to define SBP and DBP respectively. Mean blood pressure (MBP = 2/3 × DBP+1/3 × SBP) and pulse pressure (PP = SBP-DBP) were also calculated. Additional clinical parameters were also assessed, namely age, sex, body mass index (BMI) and heart rate (HR). Demographic data are in Table 2.

**Table 2:** Characteristics of the studied sample

Characteristics	Values
Sample (Female; Male)	43 (26; 17)
Clinical data, mean ± SD	
Age, years	54.1 ± 16.2
Systolic pressure, mmHg	162.7 ± 24.8
Diastolic pressure, mmHg	98.6 ± 16.6
Mean pressure, mmHg	120.0 ± 17.5
Pulse pressure, mmHg	64.0 ± 19.3
Heart rate, b/min	70.9 ± 12.4
Body mass index, kg/m^2^	27.1 ± 6.3
*Zangfu *patterns, N (%)	
Liver-fire blazing upwards	5 (11.6)
Kidney-yin deficiency and Liver-yang rising	33 (76.7)
Obstruction of phlegm and dampness of Heart/Liver/Gallbladder	0 (0)
Qi and blood deficiency leading to Liver-yang rising	0 (0)
Kidney-yin/yang deficiency	5 (11.6)
Manifestations, median [minimum; maximum]	
Presented (clinical history)	14.0 [4.0; 23.0]
Available (used in pattern differentiation)	7.0 [3.0; 10.0]

Demographic and clinical variables of subjects with hypertension

Interview with ZFHQ was performed with all subjects after clinical examination by the same Chinese medicine practitioner in the presence of another Chinese medicine doctor, each with ten years of clinical experience. The interviewer applied the ZFHQ to patients by asking them about the presence or absence of manifestations, marking them accordingly in each patient's printed questionnaire. Reports were digitized and converted to text data (string values, quoted terms and comma separated values) for analysis. Additionally, a photograph of the tongue from each patient was kept on record (Additional file 2).

### Computer-based Chinese medicine pattern differentiation

Patterns in both simulated and studied sample were identified with PDA [37,38,54]. PDA provides information of diagnostic accuracy for testing with constructed datasets. PDA performs pattern differentiation with two quantitative criteria as follows.

(1) Explained information (F_%_): calculated as the ratio between the count of manifestations found in each diagnostic hypothesis of the dataset and the total manifestations collected at the exam. This criterion indicates the 'strength' of the hypothesis as the actual diagnosis considering its predominance in the clinical history [54].

(2) Available information (N_%_): calculated as the ratio between the count of manifestations found in each diagnostic hypothesis of the dataset and the total manifestations that describe the respective diagnostic hypothesis. A cutoff point may be subtracted from the N_% _value depending on the effect of the concave-shaped curve on PDA's accuracy [38]. This criterion indicates the 'strength' of the hypothesis as the actual diagnosis considering its predominance regarding the observed hypothesis.

PDA output indicated whether or not the pattern differentiation was successful. Automatic pattern differentiation was successful if a pattern presented the highest amount of explained manifestations (F_%_) with the concomitant lowest amount of manifestations (N_%_) among two or more diagnostic hypotheses. In other words, the identified pattern maximally explained the clinical history with minimum available information. In addition to the dichotomous output (success or failure), the output comprised a nominal variable (name of identified pattern) and two continuous, percent variables (F_% _and N_% _criteria) indicating the strength of selection of pattern as a diagnostic hypothesis concerning presented and explained manifestations respectively.

### Computer-based TOD inference

The present study inferred TOD occurrence with the same method and criteria used to differentiate patterns. In this case, the output for nominal (target-organ) and percent variables (F_% _and N_%_) referred to descriptions of TOD. Likewise, F_% _and N_% _indicated the strength of inference of the target-organ as damage concerning presented and explained manifestations respectively.

### Statistical Analysis

#### Diagnostic accuracy of the questionnaire

The accuracy of ZFHQ was tested with the manifestation profile simulation algorithm (MPSA) described previously [37,38,54] for the possibility to perform accurate pattern differentiation of hypertension-related patterns using PDA. Briefly, MPSA simulated true positive cases (TP) and true negative controls (TN) manifestation profiles from ZFHD with variable amount of manifestations N_%_. The diagnosis identified by PDA was compared with the simulated condition. This process yielded a 2 × 2 confusion matrix from which binomial estimators related to diagnostic accuracy are obtained with their respective 95% confidence intervals (95%CI). The variable amount of information N_% _used by MPSA was then tested for optimum accuracy results by receiver operating curve analysis. A cutoff value for N_% _(N_%-cutoff_) was applied if a significant increase in accuracy was observed.

#### Dataset analysis

Similarity analysis between ZFHD and TODD was performed with the Jaccard coefficient *S_J _*[55] to test whether the descriptions of patterns in ZFHD were similar to descriptions of damage to target-organs in TODD. *S_J _*was in range (0;1), indicating no similarity (perfect dissimilarity) and perfect similarity respectively [56]. However, it would not be correct to infer strong similarity directly from high values of Jaccard's coefficient nor to infer weak similarity from low values because these values could be random. In turn, the random values expected to occur will depend on the number of attributes present in the sets formed by each pair of patterns and target-organs. Therefore, it was necessary to determine whether the values of Jaccard's coefficient in each pair differed from what would be expected at random in order to infer their significance. Thus, the null hypothesis was that the calculated S_J _between ZFHD and TODD was expected to occur at random and lower and upper critical values were obtained from tables [57] for acceptance of rejection of this null hypothesis considering the number of manifestations in either pattern and target-organ and the lowest number of manifestations in both pattern and target-organ descriptions. Since S_J _is a continuous variable that represent the 'strength' of association between both descriptions, it was categorized as an association measure [58], *ie *0.00 (no similarity); 0.01 to 0.20 (negligible); 0.21 to 0.40 (weak); 0.41 to 0.70 (moderate); 0.71 to 0.99 (strong); 1.00 (perfect similarity).

#### Clinical study analysis

Frequencies of manifestations among patterns and in the whole sample were tabulated. PDA was used for pattern differentiation and estimation of prevalence of each pattern in the real cases sample. Additionally, both PDA's criteria F_% _and N_% _(both continuous variables) were also calculated for the association analysis described as follows. Pearson product moment correlation was used to calculate the association between the manifestations (dichotomous variables) and hemodynamic data (continuous variables). Pearson correlation coefficient (*r*) was also used to calculate the association between patterns and TOD based on PDA's diagnostic criteria F_% _and N_% _obtained within ZFHD and TODD respectively. All candidate patterns as well as all possible target-organs output from PDA were considered simultaneously for this correlation analysis, *ie *each patient had their diagnostic criteria calculated by PDA for all *Zangfu *patterns and target-organs. Association was also categorized according to correlation coefficient [58], *ie *0.00 (no association); 0.01 to 0.20 (negligible); 0.21 to 0.40 (weak); 0.41 to 0.70 (moderate); 0.71 to 0.99 (strong); 1.00 (perfect association). Null hypotheses were *r *= 0.00 for all association tests. Statistical significance was considered at *P *< 0.05.

### Computational resources

All algorithms were implemented in LabVIEW 8.0 (National Instruments, USA) and executed on a 2.26 GHz Intel^® ^Core 2 Duo microprocessor with 2.00 GB RAM running Windows 7 (Microsoft Corporation, USA).

## Results

### Diagnostic accuracy of the questionnaire

Two hundred subjects were simulated (100 true positive [TP] and 100 true negative [TN] per pattern) by MPSA using the FM, summing up 1,000 cases. No missing cases were found (all cases presented at least one manifestation). Diagnostic accuracy with F_% _yielded the following results: TP = 481, false positive (FP) = 1, false negative (FN) = 19 and TN = 499 cases; accuracy of 98.0% [97.3; 99.1]; sensitivity and specificity of 96.2% [94.9; 98.3] and 99.8% [99.6; 100.0] respectively; negative and positive predictive values of 96.3% [95.1; 98.4] and 99.8% [99.6; 100.0] respectively. Diagnostic accuracy of PDA using F_% _and N_% _with the optimum cutoff value (19.0% of manifestations) obtained for N_% _yielded the following: TP = 487, FP = 6, FN = 13 and TN = 494 cases; accuracy of 98.1% [97.4; 99.2]; sensitivity and specificity of 97.4% [96.3; 99.2] and 98.8% [98.2; 100.0] respectively; negative and positive predictive values of 97.4% [96.4; 99.2] and 98.8% [98.1; 100.0] respectively. No significant improvement (*P *> 0.05) was found on diagnostic accuracy with the cutoff values for N_%_. Thus, no additional cutoff was applied to N_% _for pattern differentiation and TOD inference in the real patient sample.

### Similarity between ZFHD and TODD

Similarity estimated between ZFHD and TODD is in Table 3. There was a significant negligible (*S_J_≤*0.20) similarity in description between: Liver-fire blazing upwards pattern and both CVD (*S_J _= *0.15 [0.19; 0.58]) and HD (*S_J _= *0.12 [0.13; 0.86]); Obstruction of phlegm and dampness of Heart/Liver/Gallbladder pattern and KD (*S_J _= *0.11 [0.16; 0.70]); *qi *and blood deficiency leading to Liver-yang rising pattern and both HD (*S_J _= *0.00 [0.07; 0.86]) and KD (*S_J_=*0.06 [0.12; 0.86]); and Kidney-yin/yang deficiency pattern and both CVD (*S_J_=*0.15 [0.19; 0.73]) and HD (*S_J_=*0.06 [0.12; 0.86]). No significant similarity was found between any other descriptions of patterns and TOD.

**Table 3:** Similarity between descriptions of Chinese medicine patterns and target-organs damage

	Patterns				
	
Target-organs damage	Liver-fire blazing upwards (n = 11)	Kidney-yin deficiency and Liver-yang rising (n = 13)	Obstruction of phlegm and dampness of Heart/Liver/ Gallbladder (n = 10)	Qi and blood deficiency leading to Liver-yang rising (n = 7)	Kidney-yin/yang deficiency (n = 11)
**CVD** (n = 19)	0.15* [0.19; 0.58]	0.23 [0.19; 0.69]	0.26 [0.17; 0.70]	0.30 [0.15; 0.86]	0.15* [0.19; 0.73]
**HD** (n = 7)	0.12* [0.13; 0.86]	0.11 [0.11; 0.86]	0.13 [0.13; 0.86]	0.00* [0.07; 0.86]	0.06* [0.12; 0.86]
**KD** (n = 11)	0.16 [0.16; 0.72]	0.14 [0.14; 0.73]	0.11* [0.16; 0.70]	0.06* [0.12; 0.86]	0.22 [0.11; 0.73]

Jaccard coefficient of similarity between manifestations of patterns and target-organs damage calculated from ZFHD and TODD datasets.

* Values significantly lower than expected at random, *P *< 0.05. All other values not different to those expected at random were left unmarked. Values in brackets represent critical values of *S_J _*with a probability level of *P *< 0.05 considering the total number of manifestations present in either of the two patterns being compared and the minimum quantity of manifestations between patterns and target-organs. CVD: cerebrovascular and eye disease; HD: heart disease; KD: kidney disease.

### Frequency of manifestations in hypertension

Frequencies of manifestations grouped by identified hypertension-related pattern and whole sample are in Table 4. No pathognomonic manifestation was found among the whole sample of subjects with hypertension. Twelve manifestations presented high prevalence (> 50%), *ie *red tongue (81.4%); headache (72.1%); irritability (67.4%); palpitation (60.5%); blurred vision, insomnia, mental fatigue (58.1%); frequent nocturnal urination, numbness in feet and hands, shortness of breath (55.8%); and heavy limbs sensation, wiry pulse (51.2%). However, 'red tongue' was present in all five subjects with Liver-fire blazing upwards pattern while 'numbness in feet and hands' appeared in all five subjects with Kidney-yin/yang deficiency pattern.

**Table 4:** Descriptive statistics of manifestations

Manifestations	Frequency of manifestations			
	
	Liver-fire blazing upwards (n = 8; 18.6%)	Kidney-yin deficiency and Liver-yang rising (n = 30; 69.8%)	Kidney-yin/yang deficiency (n = 5; 11.6%)	Whole sample (n = 43; 100%)
red tongue	5 (100.0)	28 (84.8)	2 (40.0)	35 (81.4)
headache	2 (40.0)	26 (78.8)	3 (60.0)	31 (72.1)
irritability	2 (40.0)	27 (81.8)	0 (0)	29 (67.4)
palpitation	1 (20.0)	21 (63.6)	4 (80.0)	26 (60.5)
blurred vision	1 (20.0)	21 (63.6)	3 (60.0)	25 (58.1)
insomnia	0 (0)	23 (69.7)	2 (40.0)	25 (58.1)
mental fatigue	2 (40.0)	19 (57.6)	4 (80.0)	25 (58.1)
frequent nocturnal urination	4 (80.0)	16 (48.5)	4 (80.0)	24 (55.8)
numbness in feet and hands	0 (0)	19 (57.6)	5 (100)	24 (55.8)
shortness of breath	2 (40.0)	19 (57.6)	3 (60.0)	24 (55.8)
heavy limbs sensation	1 (20.0)	17 (51.5)	4 (80.0)	22 (51.2)
wiry pulse	4 (80.0)	17 (51.5)	1 (20.0)	22 (51.2)
tinnitus	2 (40.0)	16 (48.5)	3 (60.0)	21 (48.8)
red eyes	2 (40.0)	14 (42.4)	4 (80.0)	20 (46.5)
constipation	3 (60.0)	14 (42.4)	2 (40.0)	19 (44.2)
peeled tongue	2 (40.0)	17 (51.5)	0 (0)	19 (44.2)
numbness in the limbs	1 (20.0)	16 (48.5)	1 (20.0)	18 (41.9)
dizziness	1 (20.0)	13 (39.4)	3 (60.0)	17 (39.5)
nausea	1 (20.0)	15 (45.5)	1 (20.0)	17 (39.5)
congested feeling in the chest	1 (20.0)	13 (39.4)	2 (40.0)	16 (37.2)
weak legs	3 (60.0)	12 (36.4)	1 (20.0)	16 (37.2)
excessive dreaming	0 (0)	10 (30.3)	1 (20.0)	11 (25.6)
flushed face	1 (20.0)	8 (24.2)	2 (40.0)	11 (25.6)
thin pulse	0 (0)	9 (27.3)	2 (40.0)	11 (25.6)
impotent	1 (20.0)	7 (21.2)	2 (40.0)	10 (23.3)
strong pulse	3 (60.0)	5 (15.2)	1 (20.0)	9 (20.9)
pale tongue	0 (0)	5 (15.2)	3 (60.0)	8 (18.6)
vomiting	0 (0)	7 (21.2)	1 (20.0)	8 (18.6)
fainting	0 (0)	5 (15.2)	2 (40.0)	7 (16.3)
slippery pulse	1 (20.0)	5 (15.2)	1 (20.0)	7 (16.3)
yellow coating	3 (60.0)	4 (12.1)	0 (0)	7 (16.3)
fast pulse	1 (20.0)	4 (12.1)	0 (0)	5 (11.6)
severe dizziness	0 (0)	4 (12.1)	1 (20.0)	5 (11.6)
aphasia	0 (0)	3 (9.1)	1 (20.0)	4 (9.3)
stroke	1 (20.0)	2 (6.1)	1 (20.0)	4 (9.3)
deep pulse	0 (0)	1 (3.0)	2 (40.0)	3 (7.0)
convulsions	0 (0)	1 (3.0)	0 (0)	1 (2.3)

Frequencies of manifestations in the studied sample according to *Zangfu *patterns (manifestations were arranged in decreasing order of occurrence).

Values are shown as: absolute frequency (%).

### Association between manifestations and hemodynamic variables

SBP was weakly associated with excessive dreaming (-0.398, *P *= 0.008), shortness of breath (-0.304, *P *= 0.047) and flushed face (0.347, *P *= 0.023). DBP was also weakly associated with flushed face (0.306, *P *= 0.046) as well as to palpitation (0.355, *P *= 0.019) and to thin pulse (0.329, *P *= 0.031). MBP was weakly associated with excessive dreaming (-0.372, *P *= 0.014). PP was weakly associated with pale tongue (0.336, *P *= 0.028), and conversely with red tongue (-0.336, *P *= 0.028), strong pulse (0.304, *P *= 0.048 and shortness of breath (-0.342, *P *= 0.025). All other pairs of association were statistically no significant. After adjustment for age, sex and BMI, none of the above pairs exhibited statistically different values.

### Association between patterns and TOD

Results of the association of the diagnostic criteria between patterns and TOD are in Table 5. In respect of information F_%_, moderate association was observed between Liver-fire blazing upwards and KD (0.424; *P *= 0.004) while weak association was found between Kidney-yin deficiency and Liver-yang rising with CVD (-0.276; *P *= 0.037) and HD (-0.321; *P *= 0.019) and *qi *and blood deficiency leading to Liver-yang rising and HD (0.322; *P *= 0.019). No other comparison between pattern and TOD was significantly associated.

**Table 5:** Association between ZFHD and TODD based on diagnostic criteria

		Pattern				
	
Target-organ damage	Criterion	Liver-fire blazing upwards	Kidney-yin deficiency and Liver-yang rising	Obstruction of phlegm and dampness of Heart/Liver/ Gallbladder	Qi and blood deficiency leading to Liver-yang rising	Kidney-yin/yang deficiency
**CVD**	F_%_	-0.153	-0.276*	-0.009	0.188	-0.141
	N_%_	0.606^†^	0.637^†^	0.718^†^	0.651^†^	0.584^†^
**HD**	F_%_	-0.188	-0.321*	-0.048	0.322*	-0.263
	N_%_	0.650^†^	0.590^†^	0.700^†^	0.718^†^	0.488^§^
**KD**	F_%_	0.424^§^	0.087	0.124	-0.244	-0.096
	N_%_	0.462^§^	0.311*	0.413^§^	0.248	0.234

Pearson correlation coefficient calculated between diagnostic criteria F_% _and N_% _for each *Zangfu *pattern and target-organ in the studied sample.

*P < 0.05; ^§^*P *< 0.01; ^†^*P *< 0.001. CVD: cerebrovascular and eye disease; HD: heart disease; KD: kidney disease.

Regarding the amount of available information N_%_, Liver-fire blazing upwards pattern was moderately associated with all TOD (CVD: 0.606, *P *< 0.001; HD: 0.650, *P *< 0.001; KD: 0.462, *P *= 0.002). Kidney-yin deficiency and Liver-yang rising pattern was moderately associated with both CVD and HD (0.637, *P *< 0.001; 0.590, *P *< 0.001, respectively) and weakly associated with KD (0.311; *P *= 0.029). Obstruction of phlegm and dampness of Heart/Liver/Gallbladder pattern was strongly associated with CVD (0.718, *P *< 0.001) and moderately associated with both HD and KD (0.700, *P *< 0.001; 0.413, *P *= 0.005 respectively). *Qi *and blood deficiency leading to Liver-yang rising pattern was strongly associated with HD (0.718, *P *< 0.001) and moderately associated with KD (0.651, *P *< 0.001). Kidney-yin/yang deficiency pattern was moderately associated with CVD and HD (0.584, *P *< 0.001; 0.488, *P *= 0.001 respectively). All other comparisons yielded no significant association.

## Discussion

The main result of the present study is that *Zangfu *patterns were strongly or moderately associated with clinical manifestations of TOD in subjects with hypertension. Moreover, clinical manifestations were at most (all r < 0.40) weakly associated with hemodynamic variables.

### Relationship between Chinese medicine patterns and TOD in hypertension

The results of this study indicated that all *Zangfu *patterns were strongly (up to r = 0.718) or moderately associated with two or three target-organs due to hypertension by the amount of available information N_%_. These results were much less (up to r = 0.424) pronounced when association was tested with the explained information F_% _criterion. More interestingly, those results occurred in spite of the negligible similarity between theoretical descriptions in both ZFHD and TODD datasets. Altogether, these results indicate that the amount of information that explains a single pattern is directly proportional to the amount of information explained by the investigated target-organs for any quantity of manifestations in either clinical history or pattern. In other words, although patterns share no significant amount of manifestations with target-organs, the quantity of manifestations explained by a pattern is almost linearly proportional to the quantity of explained manifestations compatible with TOD in the same patient. The present study investigates such integrative relationship whereas other studies on morbidity of patterns in hypertension focused on descriptive statistics of patterns [27], therapeutic interventions [23-25] and proteomic analysis of dichotomous classes of patterns [26].

Chinese medicine pattern differentiation considers the presence or absence of manifestations in the exterior of the body, together with the individual constitutional characteristics, to differentiate the pattern inside the body, *ie *the internal organs and viscera. A pattern indicates the progress of a morbid condition at a certain phase, as well as the cause, nature, location, manifestation and prognosis of the condition. That is why different diseases may be associated with the same pattern and the same disease may be associated with different patterns [7-12]. The strength of this association is expected to vary with the similarity between descriptions of each pattern and disease (*ie *co-occurrence of manifestations) [38] and other factors such as relations to tissues, organs and systems functional interdependency, family history and environmental etiology [59].

In Chinese medicine, the amount of manifestations is a measure of the severity and progression of patterns [9], *ie *patterns under development are described by a small amount of manifestations (low N_% _values) while severe patterns usually presents with a large amount of manifestations (high N_% _values). The explained information criterion is based on the holistic approach that 'all manifestations must be interpreted collectively' [9,54] and thus is more influenced by co-occurrence than N_%_, which explains the strong correlation found between patterns and TOD with N_% _but not with F_%_.

By contrast, in conventional medicine, subclinical findings of diseases must be assessed with clinical and laboratorial examinations. The presence of 'silent', asymptomatic TOD (*eg *left ventricle hypertrophy, carotid atherosclerosis, diminished glomerular filtration rate, increased serum creatinine and microalbuminuria) in subjects with hypertension is already an indicator of disease progression. Silent TOD is estimated to occur in in 61.3% (any TOD) of subjects with hypertension while 50.3% of the hypertensive patients presented a single silent TOD, 31.0% two TOD and 18.7% presented three or more [60]. If left untreated, subclinical hypertension may lead to localized microvascular lesions (atherosclerosis) which can progress into diffuse (arteriosclerosis) lesions, affecting target-organs and producing various manifestations [32], *ie *silent TOD slowly progresses to symptomatic TOD. Because of the progression of structural damages, hypertension can be undiscovered for 10-20 years [61] and the overall prevalence of clinically manifested TOD can be as high as 95% for stroke (CVD), 89% for left ventricular hypertrophy (HD) and 95% for kidney failure (KD) [62]. Although this study does not present data regarding silent TOD to guarantee that patients actually present any degree of TOD - in fact, for some manifestations is not quite necessary (*eg *stroke) - the collective results of this first study strongly indicate that the five *Zangfu *patterns commonly used for pattern differentiation in patients with hypertension are indeed related to hypertension-induced TOD. Further studies should focus on subclinical findings in hypertension and their relationship with symptomatic hypertensive patients and Chinese medicine patterns.

### Frequency distribution of patterns and manifestations

The frequency distribution of patterns observed in this sample is in agreement with previously studies. Maklin *et al*. [24] reported that Kidney-yin deficiency and Liver-yang rising (*shen yin xu gan yang shang yan*) was the most prevalent pattern (47-63%), followed by obstruction of phlegm and dampness of Heart/Liver/Gallbladder (*xin gan dan shi tan bi*) (19-30%), Liver-fire blazing upwards (*gan huo shang yan*) (13-17%), *qi *and blood deficiency leading to Liver yang rising (*qi xue xu gan yang shang yan*) (2-6%) and Kidney-yin/yang deficiency (*shen yin yang xu*) (0-3%). Gu *et al*. [27] found stagnation of phlegm-dampness (*zhi shi tan*) to be the most prevalent pattern (27%), followed by hyperactivity of the Liver-yang (*gan yang shang yan*) (24%), deficiency of Heart/Kidney-Qi (*xin shen qi xu*) (10%), blood stasis obstructing the collaterals (*luo xue yu bi*) (9%), deficiency of yin and yang (*yin yang xu*) (8%) while other syndromes accounted for 21% of the sample. Why patterns related to Liver-yang and phlegm-dampness are the most prevalent is still unknown. Emotional states, family history and food habits play important roles in the etiology of these patterns and are considered as major risk factors to hypertension by both Chinese medicine [9,11] and conventional medicine [29].

### Association between manifestations, patterns, and hemodynamic data

High blood pressure levels should be symptomless; however, patients and physicians usually attribute symptoms to increased levels of blood pressure. The weak significant association between manifestations and blood pressure variables observed in this study were not held under adjustment for age, sex and BMI. These results agree with the physiologic knowledge on blood pressure control and with other epidemiologic reports according to which symptoms were not significantly correlated to hypertension [63,64], patterns [27] or proved uncorrelated when adjusted to confounding variables [65] or awareness of hypertension [66]. In the present study, association tests were performed with the entire sample of hypertensive subjects. It is possible that predominance of Kidney-yin deficiency and Liver-yang rising pattern lead to biased results. Further studies may search for such correlations with the sample divided into equally distributed subgroups regarding all identified patterns and subsample sizes.

### Using manifestations to bridge the gap between Chinese medicine and conventional medicine

The present study regards the patient as the common element to both medical practices. Chinese medicine practitioners and physicians interpret clinical manifestations according to their medical training and may not rely on information provided by laboratories and medical imaging. Conventional medicine considers the manifestations of hypertension-induced TOD as consequences of progressive, structural lesions to arteries that progressively compromise blood flow to and cell metabolism of vital organs. Chinese medicine interprets the same manifestations as due to chronic, functional imbalances of organs and viscera that result in *Zangfu *deficiency states and obstruction or rebellion of *qi*, yin, yang or blood (*xue*). Risk factors for hypertension are quite identical in these two medical systems and stress the observed strong association between TOD with manifestations and *Zangfu *patterns.

### Implications for proper antihypertensive agents selection

While current pharmacological treatment for hypertension is based on the level of SBP and DBP and the level of total cardiovascular risk [29], however, Chinese medicine diagnosis with disease subtyping may provide insights into optimization of classes of antihypertensive medications for TOD management. For instance, research suggests that specific agents work better in treating hypertension with particular patterns, *eg *calcium channel blockers for phlegmatic damp excess pattern and blood stasis; β-blockers for liver-yang rising; angiotensin converting enzyme inhibitors for yin-deficiency and yang-hyperactivity or combined liver-yin and kidney-yin deficiency [22]. The therapeutic potential of several antihypertensive agents (diuretics, angiotensin converting enzyme inhibitors, angiotensin II receptor antagonists, β-blockers, calcium channel blockers and aldosterone antagonism) have been shown to also improve hypertension-induced TOD [32]. However, the efficacy of antihypertensive agents, acupuncture and herbs as well as the effects of such interventions on TOD is yet to be determined.

## Conclusion

*Zangfu *patterns are associated with clinical manifestations of TOD. Manifestations associated patterns indicate morbid conditions to be secondary to hypertension rather than simple blood pressure.

## Abbreviations

95%CI: 95% confidence interval; BMI: body mass index; CVD: cerebrovascular and eye disease; DBP: diastolic blood pressure; F_%_: proportion of explained information of pattern from clinical history; FM: Four methods; FN: false negative; FP: false positive; HD: heart disease; HR: heart rate; KD: kidney disease; MBP: mean blood pressure; MPSA: manifestation profile simulation algorithm; N_%_: proportion of available information of pattern in dataset; N_%-cutoff_: proportion of optimized available information of pattern in dataset; PDA: pattern differentiation algorithm; PP: pulse pressure; *r*: Pearson correlation coefficient; SBP: systolic blood pressure; S_J_: Jaccard coefficient of similarity; STROBE: Strengthening the Reporting of Observational Studies in Epidemiology; TN: true negative; TOD: target-organ damage; TODD: target-organ disease dataset; TP: true positive; ZFHD: *Zangfu *hypertension dataset; ZFHQ: *Zangfu *hypertension questionnaire.

## Competing interests

The authors declare that they have no competing interests.

## Authors' contributions

ASF designed the study, developed the computational methods for pattern differentiation, performed the statistical analysis and drafted the manuscript. ABL performed literature review and questionnaire interview. JBF and IC evaluated and diagnosed the patients for enrollment in the study. All authors revised and approved the final version of the manuscript.

## Supplementary Material

Additional file 1 ***Zangfu *patterns hypertension dataset (ZFHD)**. This table presents the complete description of manifestations regarding *Zangfu *patterns and distributed among the Examination methods Click here for file

Additional file 2 **Representative cases for each *Zangfu *pattern in hypertension**. This table presents a representative case for each diagnosis, including its manifestations and tongue pictures for illustration of cases. Click here for file
