# Supplementary material for: Zangfu zheng (patterns) are associated with clinical manifestations of zang shang (target-organ damage) in arterial hypertension
Source: Chin Med. 2011 Jun 17;6:23. doi: 10.1186/1749-8546-6-23 (PMC3155491; doi:10.1186/1749-8546-6-23)
Supplement: Additional file 1 — Zangfu patterns hypertension dataset (ZFHD). This table presents the complete description of manifestations regarding Zangfu patterns and distributed among the Examination methods [file 1749-8546-6-23-S1.PDF]

***Zangfu* patterns hypertension dataset (ZFHD).**

This table presents the complete description of manifestations regarding *Zangfu* patterns and distributed among the Examination methods.

Examination method	*Zangfu* pattern				
	**Liver-fire blazing upwards**	**Kidney-yin deficiency and Liver-yang rising**	**Obstruction of phlegm and dampness of Heart/ Liver/ Gallbladder**	**Qi and blood deficiency leading to Liver-yang rising**	**Kidney-yin/ yang deficiency**
Inspection	red togue, red eyes, red face, yellow coating	peeled tongue, red tongue	grease and thick coating	*	pale tongue
Auscultation-Olfaction	*	*	*	aphasia	shortness of breath
Inquiry	constipation headache, irritability, tinnitus	headache, numbness in the limbs, insomnia, irritability, excessive dreaming, tinnitus, dizziness, blurred vision	numbness in the limbs, nausea, palpitation, congested feeling in the chest, heavy limbs sensation, dizziness, vomiting	stroke, convulsions, headache, severe dizziness	mental fatigue, numbness in feet and hands, weak legs, impotent, frequent nocturnal urination, tinnitus, dizziness
Palpation	wiry pulse, strong pulse, fast pulse	wiry pulse, thin pulse, fast pulse	wiry pulse, slippery pulse	wiry pulse	deep pulse, thin pulse

* Not reported in the consulted literature.
