# Supplementary material for: Zangfu zheng (patterns) are associated with clinical manifestations of zang shang (target-organ damage) in arterial hypertension
Source: Chin Med. 2011 Jun 17;6:23. doi: 10.1186/1749-8546-6-23 (PMC3155491; doi:10.1186/1749-8546-6-23)
Supplement: Additional file 2 — Representative cases for each Zangfu pattern in hypertension. This table presents a representative case for each diagnosis, including its manifestations and tongue pictures for illustration of cases. [file 1749-8546-6-23-S2.PDF]

Representative cases for each *Zangfu* pattern in systemic arterial hypertension.

Diagnosis, manifestations, tongue pictures were provided for illustration of cases. Manifestations explained by the respective identified pattern are marked bold.

Representative case	Identified pattern	Manifestations	Tongue picture
Case #10	Liver-fire blazing upwards	"**headache**", "mental fatigue", "**irritability**", "**red tongue**", "frequent nocturnal urination", "**wiry pulse**", "**strong pulse**", "**yellow coating**", "**tinnitus**"	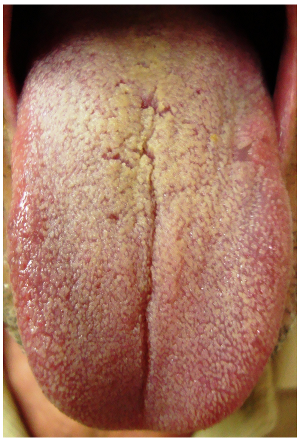
Case #5	Kidney-yin deficiency and Liver-yang rising	"**numbness in the limbs**", "numbness in feet and hands", "weak legs", "mental fatigue", "impotent", "**insomnia**", "**irritability**", "**peeled tongue**", "**red tongue**", "frequent nocturnal urination", "nausea", "**wiry pulse**", "strong pulse", "shortness of breath", "**tinnitus**", "severe dizziness", "**blurred vision**"	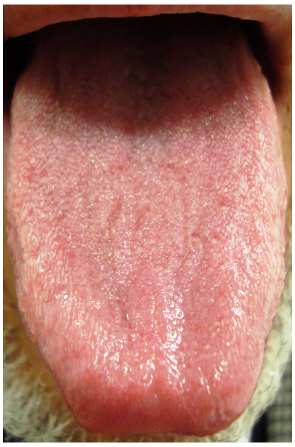
Case #29	Kidney-yin/yang deficiency	"**pale tongue**", "red eyes", "**shortness of breath**", "headache", "**numbness in feet and hands**", "insomnia", "**frequent nocturnal urination**", "palpitation", "**tinnitus**", "**dizziness**", "blurred vision", "**deep pulse**"	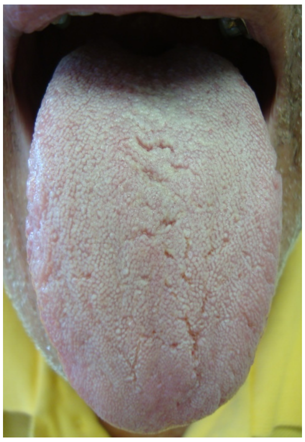
